# Non-Isothermal Oxidation Behaviors and Mechanisms of Ti-Al Intermetallic Compounds

**DOI:** 10.3390/ma12132114

**Published:** 2019-06-30

**Authors:** Peixuan Ouyang, Guangbao Mi, Peijie Li, Liangju He, Jingxia Cao, Xu Huang

**Affiliations:** 1 Aviation Key Laboratory of Science and Technology on Advanced Titanium Alloys, AECC Beijing Institute of Aeronautical Materials, Beijing 100095, China; 2 National Center of Novel Materials for International Research, Tsinghua University, Beijing 100084, China; 3 School of Mechanical and Materials Engineering, North China University of Technology, Beijing 100144, China; 4 Beijing Engineering Research Center of Graphene and Application, Beijing 100095, China

**Keywords:** titanium aluminides, oxidation, non-isothermal, mechanism, internal oxidation

## Abstract

Non-isothermal oxidation is one of the important issues for the safe application of Ti-Al alloys, so this study aimed to illustrate the non-isothermal oxidation behaviors and the corresponding mechanisms of a TiAl-based alloy in comparison with a Ti_3_Al-based alloy. The non-isothermal oxidation behaviors of Ti-46Al-2Cr-5Nb and Ti-24Al-15Nb-1.5Mo alloys in pure oxygen were comparatively investigated with a thermogravimetry-differential scanning calorimetry (TGA/DSC) simultaneous thermal analyzer heating from room temperature to 1450 °C with a heating rate of 40 °C/min. When the temperature rose above 1280 °C, the oxidation rate of the Ti-46Al-2Cr-5Nb alloy sharply increased and exceeded that of the Ti-24Al-15Nb-1.5Mo alloy owing to the occurrence of internal oxidation. When the temperature was higher than 1350 °C, the oxidation rate of the Ti-46Al-2Cr-5Nb alloy decreased obviously due to the generation of an oxygen-barrier β-Al_2_TiO_5_-rich layer by a chemical reaction between Al_2_O_3_ and TiO_2_ in the oxide scale. Based on Wagner’s theory of internal oxidation, the reason for the occurrence of internal oxidation in the Ti-46Al-2Cr-5Nb alloy is the formation of the α phase in the subsurface, while no internal oxidation occurred in the Ti-24Al-15Nb-1.5Mo alloy due to the existence of the β phase in the subsurface with the enrichment of Nb and Mo.

## 1. Introduction

TiAl-based alloys have received considerable attention as high-temperature structural materials for aerospace and automotive applications, since they maintain numerous outstanding properties, such as low density (3.9–4.2 g/cm^3^), high specific strength, good creep resistance and excellent fireproof performance [1,2,3,4,5]. Nevertheless, the insufficient high-temperature oxidation resistance of the TiAl-based alloys limits their wide application and development [6,7]. Numerous investigations have been carried out to study the high-temperature oxidation behaviors of the TiAl-based alloys, most of which were concerned about the long-term isothermal oxidation at normal service temperature (800–1000 °C) [8,9,10]. Few studies have been focused on the non-isothermal oxidation behaviors of the TiAl-based alloys, which is also deserving of attention since the alloys are often subject to non-isothermal oxidation in service. For example, aero-engine TiAl components are heated rapidly from room temperature to service temperature during the engine startup period. For another instance, aero-engine TiAl blades are likely to suffer from titanium fire, which consists of ignition and propagation combustion processes under the induction of external energies such as high-energy friction, fracture, melt droplets and so on [11], even though the fireproof performance of TiAl-based alloys is much better than that of conventional titanium alloys. Ignition, which is the precursor process of titanium fire, is essentially a non-isothermal oxidation process [12,13] and the ignition points of titanium alloys are slightly lower than their melting points [11]. Hence, the non-isothermal oxidation of TiAl-based alloys with a rather wide temperature range has a major impact on their safe application in aeroengines.

In addition, the non-isothermal oxidation behaviors of the TiAl-based alloys should be more special than the isothermal oxidation behaviors, since the heating rate is so high that the oxidation may not reach dynamic equilibrium and the temperature range is so wide that phase transition may occur in the alloys. Many studies showed that oxidation temperature and time significantly affect isothermal oxidation behaviors of the TiAl-based alloys [14,15,16,17]. Moreover, Vaidya [18] found that during isothermal oxidation, the mass gain of Ti-48Al-2Nb-2Cr alloy exceeds that of Ti-25Al-10Nb-3V-1Mo alloy when the temperature is above 1200 °C, which breaks away from the general understanding that the oxidation resistance of the TiAl-based alloys is better than that of the Ti_3_Al-based alloys. This phenomenon is considered to be attributed to the doubly increased oxygen solubility of γ phase in the TiAl-based alloy at 1200 °C [18], while this explanation seems unreasonable since the maximum oxygen solubility of γ phase (~3 at.%) in the TiAl-based alloy is much lower than that of α_2_ phase (~13 at.%) in the Ti_3_Al-based alloys [19]. Hence, in regards to non-isothermal oxidation, it should be of interest to people what behaviors the TiAl-based alloys would exhibit, whether there exists a similar phenomenon that the oxidation rate of the TiAl-based alloys exceeds that of the Ti_3_Al-based alloys at high temperature, as well as what is the reasonable explanation for this phenomenon. On the basis of the above questions, this paper examines the non-isothermal oxidation behaviors of a TiAl-based alloy in comparison with a Ti_3_Al-based alloy and illustrates the corresponding oxidation mechanisms. On one hand, this research helps to better understand the ignition mechanisms of TiAl alloys, on the other hand, it might promote the development of new-type TiAl alloys resistant to higher temperature and with less risk to titanium fire by ingredient optimum design.

TGA/DSC simultaneous thermal analysis is a common method to study the non-isothermal oxidation behaviors of metals and their alloys [20,21,22,23]. Adopting the TGA/DSC method, G.B. Mi et al. [22] studied the effect of Cr content on the non-isothermal oxidation behaviors of Ti-Cr fire-proof titanium alloys. The results showed that when the Cr content exceeds 10–15 wt.%, the oxidation resistance of Ti-Cr alloys increases with the Cr content due to the precipitation of Cr oxide [22]. Ouyang et al. [23] carried out a research on the non-isothermal oxidation behaviors of a high-temperature near-α titanium alloy (TA29 alloy), meanwhile discussed the effects of lattice transformation and alloying elements on the non-isothermal oxidation behaviors of the TA29 alloy.

In this paper, the non-isothermal oxidation behaviors of a TiAl-based alloy and a Ti_3_Al-based alloy in pure oxygen were studied with a TGA/DSC synchronous thermal analyzer heating from room temperature to 1450 °C with a heating rate of 40 °C/min. Combined with microstructural characterization and calculation of oxidation activation energy, the non-isothermal oxidation mechanisms of the TiAl-based alloy were elucidated. Furthermore, the reason for the poorer oxidation resistance of the TiAl-based alloy than that of the Ti_3_Al-based alloy at high temperature was revealed based on Wagner’s theory of internal oxidation.

## 2. Experimental

### 2.1. Specimen Preparation

**Figure 1:**
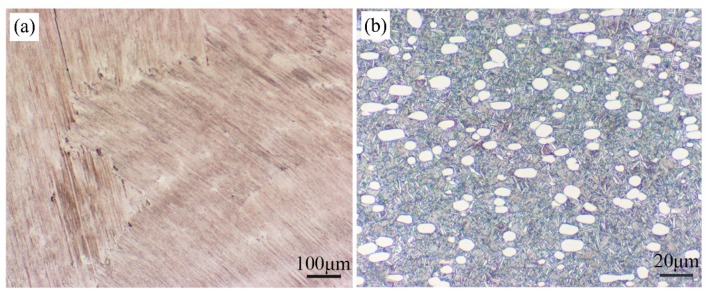
Optical micrographs showing the original microstructure of the (**a**) Ti-46Al-2Cr-5Nb and (**b**) Ti-24Al-15Nb-1.5Mo alloys.

The nominal compositions of the TiAl-based and Ti_3_Al-based alloys studied in this paper are Ti-46Al-2Cr-5Nb (at.%) and Ti-24Al-15Nb-1.5Mo (at.%), respectively. Both the two alloys were prepared by melting, forging, heat treatment and mechanical processing. The microstructure of the as-received Ti-46Al-2Cr-5Nb alloy consists of coarse fully-lamellar γ + α_2_ colonies, as shown in Figure 1a. The microstructure of the as-received Ti-24Al-15Nb-1.5Mo alloy is duplex, being composed of equiaxed primary α_2_ and/or O phases, B2 transformed structure consisting of flaky secondary α_2_ and O phases and residual B2 phase, as shown in Figure 1b. For non-isothermal oxidation experiments, specimens with dimensions of 3 × 2 × 2 mm^3^ were cut from the two kinds of alloy sheets by a computerized numerical control (CNC) dicing saw (SYJ-400, Shenyang kejing instrument company, Shenyang, China. The specimens were ground with sandpapers up to 2000-grit to remove oxide scales and then ultrasonically cleaned with acetone and alcohol.

### 2.2. Non-Isothermal Oxidation Experiment

Non-isothermal oxidation experiments were carried out in a TGA/DSC simultaneous thermal analyzer (TGA/DSC 1; Mettler Toledo, Zurich, Switzerland). The specimens of both the Ti-46Al-2Cr-5Nb and Ti-24Al-15Nb-1.5Mo alloys were heated from room temperature to 1450 °C at a heating rate of 40 °C/min under a pure oxygen flow of 50 mL/min. The oxygen flow was added to the system once the heating process was started and the flow was cut off when the heating process was completed. After furnace cooling the oxidized specimens were taken out. For each alloy, the mass-gain, mass-gain rate, and heat flux curves were obtained from three repeated experiments with the relative standard deviations less than 10%. Besides, in order to investigate the non-isothermal oxidation behaviors of the two alloys in detail, the specimens were heated to several interested temperatures below 1450 °C and then were furnace cooled to obtain the corresponding non-isothermal oxidation products.

### 2.3. Microstructural Characterization

Specimens were examined after non-isothermal oxidation using field emission scanning electron microscopy (FE-SEM; Hitachi SU8000, Tokyo, Japan) and X-ray diffraction (XRD; Bruker D8 Advance; Cu Ka, Karlsruhe, Germany) to characterize surface morphologies of the oxide scales and identify oxide phases. For cross-sectional microstructure observation of the oxidized specimens, metallographic specimens were prepared by being placed in the transverse direction, being mounted with cold-setting resin, then being ground with 400–2000 grit SiC sandpapers, being polished with 1.0 μm alumina suspension, being etched with Kroll reagent (92 mL H_2_O, 3 mL HF, 5 mL HNO_3_) and finally being coated with a thin layer of carbon. The microstructures and elemental distributions on the cross-section of the oxide scales were characterized using an electron probe microanalyzer (EPMA; Shimadzu EPMA-1720H, Kyoto, Japan).

## 3. Calculation of Oxidation Activation Energy

Similar to traditional titanium alloys, the oxidation mass gains of the Ti-Al alloys also come from oxygen dissolution and growth of oxide scales. The two oxidation behaviors are competitive and one of them governs the mass gain at a certain temperature and time [24,25,26]. The rate-determining steps of the two oxidation behaviors are respectively the diffusion of O atom in the alloy and the diffusion of O^2−^ in the oxide scale. Thus, it can be assumed that the mass-gain rate of the Ti-Al alloys during non-isothermal oxidation is limited by one-dimensional diffusion of one species A (A is O atom or O^2−^), so that the mass-gain rate per unit area of the Ti-Al alloys is proportional to the molar diffusion flux of specie A. Furthermore, based on the Fick’s diffusion law and assuming that the diffusion of specie A satisfies Arrhenius kinetics, the relationship among the temperature, the mass gain and the oxidation activation energy of the Ti-Al alloys can be derived, as shown in Equation (1). Details for the derived process can be seen in our previous work [23].

(1) $$-\ln\frac{d(\Delta m)}{dT}-\ln(\Delta m)=-\ln K^{*}+E/RT$$

where Δ*m* is the mass gain per unit area, *E* is the oxidation activation energy, R is the molar gas constant and K* is constant. Let the left side of Equation (1) be equal to *Y*(Δ*m*), thus *Y*(Δ*m*) is linearly related to the reciprocal of temperature and the product of the corresponding positive slope and R is the oxidation activation energy.

## 4. Results and Discussion

### 4.1. Oxidation Mass Gain and Activation Energies

**Figure 2:**
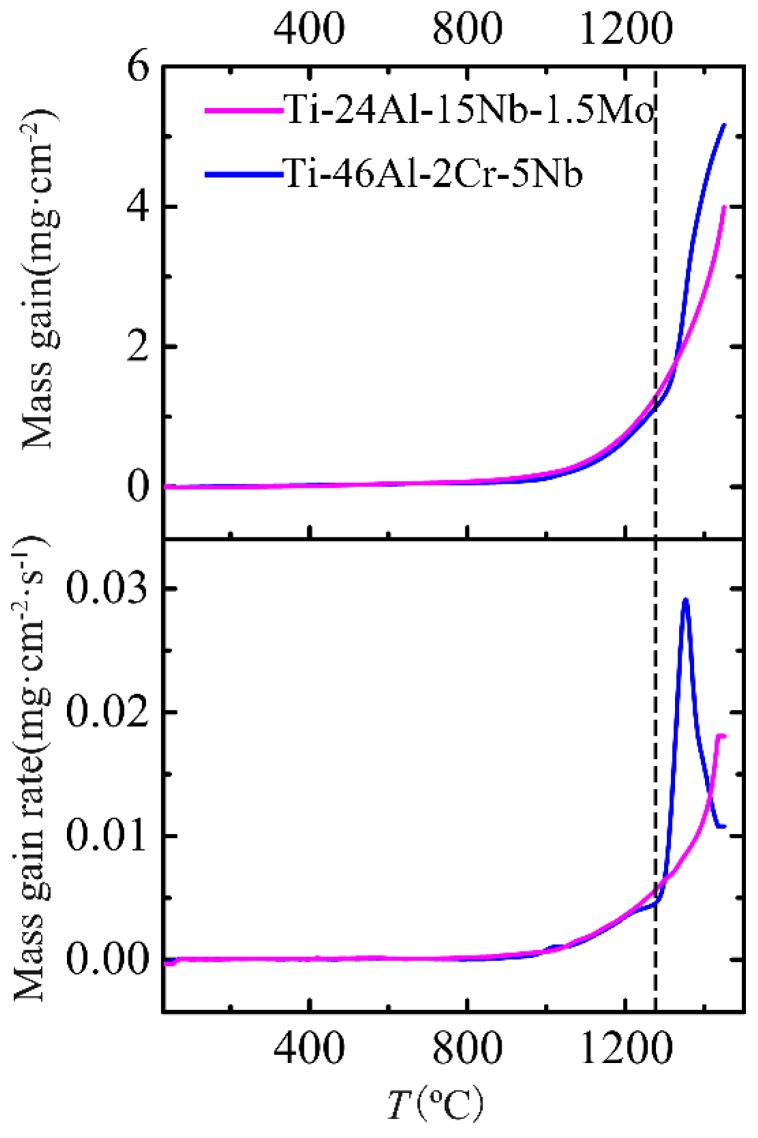
Comparisons of the mass-gain and mass-gain rate curves between the Ti-46Al-2Cr-5Nb and Ti-24Al-15Nb-1.5Mo alloys obtained during non-isothermal oxidation.

Figure 2 shows the mass-gain and mass-gain rate curves of the Ti-46Al-2Cr-5Nb and Ti-24Al-15Nb-1.5Mo alloys obtained during non-isothermal oxidation. The mass-gain rates of the two alloys were similar when the temperature was lower than 1280 °C. However, the mass-gain rate of the Ti-46Al-2Cr-5Nb alloy increased dramatically when the temperature was higher than 1280 °C, resulting that the mass gain of the Ti-46Al-2Cr-5Nb alloy significantly exceeded that of the Ti-24Al-15Nb-1.5Mo alloy. This phenomenon is not common but is similar to that reported by Vaidya [18]. Nevertheless, the mass-gain rate of the Ti-46Al-2Cr-5Nb alloy turned to decrease obviously when the temperature was above 1350 °C.

**Figure 3:**
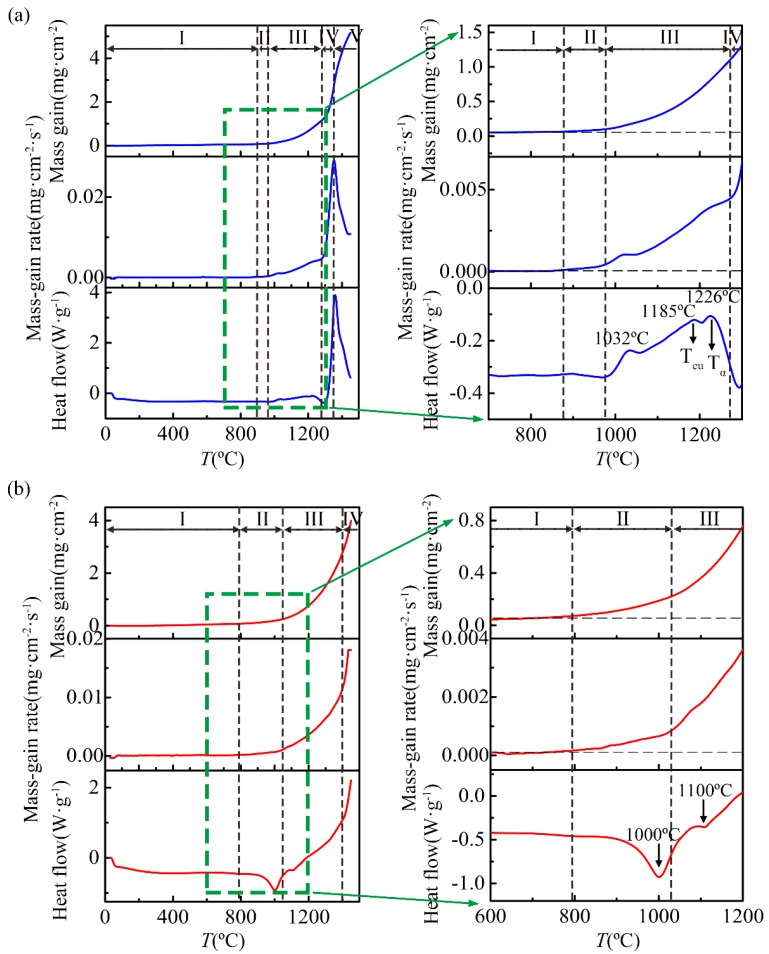
Stage divisions of the non-isothermal oxidation process for the (**a**) Ti-46Al-2Cr-5Nb and (**b**) Ti-24Al-15Nb-1.5Mo alloys according to the evolution of the mass-gain rates.

According to the evolution of the mass-gain rate, the non-isothermal oxidation process of the Ti-46Al-2Cr-5Nb alloy could be divided into five stages for further study, as shown in Figure 3a. When the temperature was below 870 °C (Stage I), the mass gain of the Ti-46Al-2Cr-5Nb alloy was few and could be neglected. When the temperature rose to 870–980 °C (Stage II), the mass-gain rate increased slowly and the corresponding mass gain was 0.04 mg/cm^2^. When the temperature was increased to 980–1280 °C (Stage III), the mass-gain rate increased obviously and the corresponding mass gain was 1.05 mg/cm^2^. When the temperature was raised to 1280–1350 °C (Stage IV), the mass-gain rate increased sharply and the corresponding mass gain was 1.68 mg/cm^2^. Nevertheless, the mass-gain rate decreased significantly when the temperature was above 1350 °C (Stage V). It is concluded that the non-isothermal oxidation process of the Ti-46Al-2Cr-5Nb alloy consists of five stages, including nearly non-oxidation (Stage I, <870 °C), slow oxidation (Stage II, 870–980 °C), accelerated oxidation (Stage III, 980–1280 °C), severe oxidation (Stage IV, 1280–1350 °C) and decelerated oxidation (Stage V, 1350–1450 °C) stages. However, the non-isothermal oxidation process of the Ti-24Al-15Nb-1.5Mo alloy can be only divided into four stages according to the evolution of the mass-gain rate, as shown in Figure 3b, including nearly non-oxidation (Stage I, <800 °C), slow oxidation (Stage II, 800–1020 °C), accelerated oxidation (Stage III, 1020–1400 °C) and severe oxidation (Stage IV, 1400–1450 °C) stages. Table 1 gives a summary of the temperature ranges corresponding to the different stages of the non-isothermal oxidation process for the Ti-46Al-2Cr-5Nb and Ti-24Al-15Nb-1.5Mo alloys.

**Figure 4:**
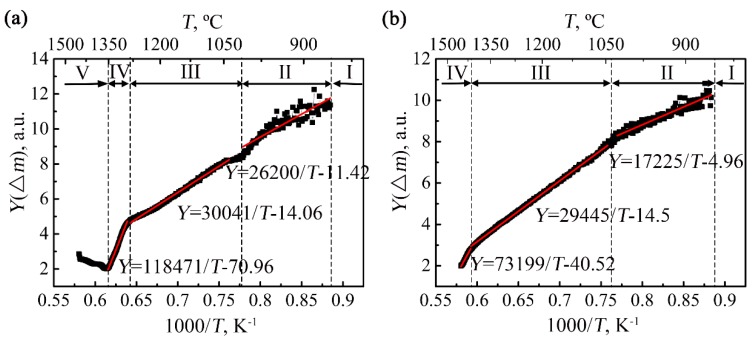
The *Y*(∆*m*)~1/*T* curves and the corresponding linear fitting results of the (**a**) Ti-46Al-2Cr-5Nb and (**b**) Ti-24Al-15Nb-1.5Mo alloys.

Figure 4 presents the *Y*(Δ*m*)~1/*T* curves and the corresponding linear fitting results of the two alloys. The evolutions of the slops of the *Y*(Δ*m*)~1/*T* curves also clearly demonstrate that the non-isothermal oxidation processes of the Ti-46Al-2Cr-5Nb and Ti-24Al-15Nb-1.5Mo alloys are respectively composed of five stages and four stages. It should be noted that since the mass changes of the two alloys at Stage I were mainly induced by the small mass fluctuation of the thermogravimetric analysis equipment, the values of *Y*(Δ*m*) at Stage I fluctuated around a certain value and are not shown in Figure 4. Besides, the *Y*(Δ*m*)~1/*T* curves of both the two alloys at Stages II, III and IV could be positive linearly fitted, so that the oxidation activation energies of the two alloys for the three stages were obtained, as presented in Table 1. It can be seen that the oxidation activation energies of the two alloys at Stage III are similar, while the oxidation activation energies of the two alloys both at Stage II and at Stage IV are quite different, which is related to their oxidation mechanisms and will be discussed in Section 4.3.

### 4.2. Matrix Phases

Since the non-isothermal oxidation experiments were carried out in a wide temperature range, the Ti-46Al-2Cr-5Nb and Ti-24Al-15Nb-1.5Mo alloys were likely to undergo phase transitions during non-isothermal oxidation.

Generally, the phase transition types and temperatures of the TiAl-based alloys are affected not only by the aluminum content (45–48 at.%), but also by kinds of other alloying elements (such as Cr, V, Mn, Nb, Ta, W, Si, C, P and B) and their contents (0.1–8 at.%) [27]. According to the Ti-Al phase diagram [28], the heat-flow and thermomechanical analysis (TMA) derivative curves of Ti-(46~47)Al-2Cr-(2~8)Nb alloy [29], the phase transitions of the Ti-46Al-2Cr-5Nb alloy heating from room temperature to 1450 °C are deduced to be γ + α_2_ → γ + α and γ + α → α with the respective temperature range of 1175–1215 °C (*T*_eu_) and 1292–1295 °C (*T*_α_). The deduced temperature ranges are in accordance with the two exothermic peaks with the respective temperatures of 1185 and 1226 °C in the heat-flow curve of the Ti-46Al-2Cr-5Nb alloy, as shown in Figure 3a. It confirms that the phase transitions of γ + α_2_ → γ + α and γ + α → α occurred in the Ti-46Al-2Cr-5Nb alloy during the non-isothermal oxidation. Besides, it can be seen from Figure 3a that there exists another exothermic peak with a temperature of about 1032 °C in the heat-flow curve of the Ti-46Al-2Cr-5Nb alloy. It is not yet possible to explain the physical meaning of this peak based on the Ti-Al phase diagram. However, it has been reported that there exists a similar exothermic peak in the heat-flow curve of Ti-46Al-1.9Cr-3Nb alloy with typical duplex structure, which is ascribed to equilibrium transformation or uniformity when heating of the non-equilibrium structure generated by thermal mechanical processing [30]. Therefore, the matrix phases of the Ti-46Al-2Cr-5Nb alloy at different stages of the non-isothermal oxidation process could be concluded, as shown in Table 1.

As for the Ti-24Al-15Nb-1.5Mo alloy, the Nb equivalent is about 20 at.% since the beta phase stability of Mo is 3.6 times of that of Nb [31]. Thus, according to the Ti-25Al-Nb phase diagram [31], the phase transitions of the Ti-24Al-15Nb-1.5Mo alloy heating from room temperature to 1450 °C are deduced to be α_2_ + O + B2 → α_2_ + B2 (1010 °C), α_2_ + B2 → B2 (1100 °C) and B2 → β (1220 °C). Since there exist two endothermic peaks with the respective temperatures of near 1000 and 1100 °C in the heat-flow curve of the Ti-24Al-15Nb-1.5Mo alloy during non-isothermal oxidation, as shown in Figure 3b, it demonstrates the occurrence of the former two phase transitions. Besides, no peak corresponding to the last phase transition could be found in the heat-flow curve of the Ti-24Al-15Nb-1.5Mo alloy, which is probably due to the small chemical heat for the disordering from B2 phase to β phase. As a result, the matrix phases of the Ti-24Al-15Nb-1.5Mo alloy at different stages of the non-isothermal oxidation process could be also concluded, as presented in Table 1.

### 4.3. Non-Isothermal Oxidation Mechanisms

#### 4.3.1. Nearly Non-Oxidation Stage (Stage I)

Similar to the near-α titanium alloy TA29 [23], the oxidation mass gains of the Ti-46Al-2Cr-5Nb and Ti-24Al-15Nb-1.5Mo alloys at Stage I were very small and could be neglected (Figure 3), which is ascribed to the fact that a thin titanium oxide film (200 nm) rapidly forms on the alloys at room temperature and passivates the surface [14].

#### 4.3.2. Slow Oxidation Stage (Stage II)

When the temperature rose to Stage II (870–980 °C for the Ti-46Al-2Cr-5Nb alloy and 800–1020 °C for the Ti-24Al-15Nb-1.5Mo alloy), both the two alloys exhibited slow oxidation behaviors, which is also similar to the near-α titanium alloy TA29 at Stage II with the temperature range of 750–1000 °C [23]. As discussed in our previous work [23], the oxidation mechanism of the TA29 alloy at Stage II is oxygen dissolution with the rate-determining step of oxygen diffusion in the alloy. The oxidation activation energies of the TA29 alloy at Stage II (163.9 kJ/mol) [23] is about 20 kJ/mol higher than that of the Ti-24Al-15Nb-1.5Mo alloy (143.2 kJ/mol). The matrixes of the TA29 and Ti-24Al-15Nb-1.5Mo alloys at Stage II are respectively dominated by the α and α_2_ phases. Moreover, it has been reported that the activation energy for oxygen diffusion in the α phase is 10–20 kJ/mol higher than that in the α_2_ phase [32], which is equivalent to the difference of the oxidation activation energy between the TA29 and Ti-24Al-15Nb-1.5Mo alloys. Therefore, the oxidation mechanism of the Ti-24Al-15Nb-1.5Mo alloy at Stage II is also the oxygen dissolution in the alloy. It indicates that the thin titanium oxide film (200 nm) passivating the surface at Stage I could not prevent oxygen from diffusing into the alloys at Stage II, which might be due to the increase of the solubility of TiO_2_ in the alloys or the cracking of TiO_2_ film caused by phase transitions in the oxide film with the temperature increases.

**Figure 5:**
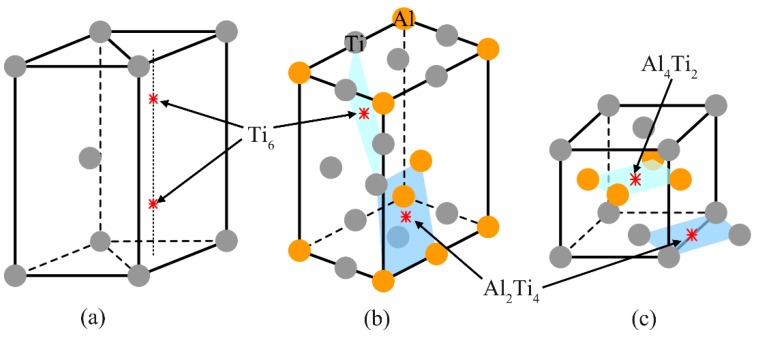
Octahedral interstitial sites for oxygen atoms in the lattices of the (**a**) α, (**b**) α_2_ and (**c**) γ phases.

The lower oxidation activation energy of the Ti-24Al-15Nb-1.5Mo alloy in comparison with the TA29 alloy indicates that oxygen atoms diffuse much easier in the former alloy. Since volume diffusion is the main diffusion type in the alloys at such high temperature, the difference of oxygen diffusion ability can be explained by the difference of the lattice structure between the two alloys. The α phase dominating in the TA29 alloy has the close-packed hexagonal structure (hcp, A3), where oxygen atoms tend to occupy the two Ti_6_ octahedral interstitial sites [33,34], as shown in Figure 5a. The α_2_ phase dominating in the Ti-24Al-15Nb-1.5Mo alloy has the ordered close-packed hexagonal structure (DO_19_), where oxygen atoms prefer to occupy the Ti_6_ octahedral interstitial site instead of the Al_2_Ti_4_ site [35,36], as shown in Figure 5b. Though the number of the Ti_6_ interstitial site in the lattice of the α_2_ phase is lower than that in the lattice of the α phase, which leads to the lower oxygen solubility in the α_2_ phase than in the α phase, the covalence of Ti-Al bond in the lattice of the α_2_ phase makes electrons aggregate between Ti and Al atoms, resulting in the weaker Ti-O bond strength in the lattice of the α_2_ phase compared with that in the lattice of the α phase [32]. Therefore, the diffusion resistance of oxygen atoms in the lattice of the α_2_ phase is smaller than that in the lattice of the α phase. That is to say, the required activation energy for oxygen diffusion in the α_2_ phase is smaller than in the α phase.

As for the Ti-46Al-2Cr-5Nb alloy, the oxidation activation energy at Stage II (217.8 kJ/mol) is higher than that of the Ti-24Al-15Nb-1.5Mo alloy (143.2 kJ/mol). The γ phase dominating in the Ti-46Al-2Cr-5Nb alloy at Stage II has the ordered face-centered cubic structure (L_10_), where oxygen atoms can only occupy the interstitial octahedral sites surrounded by both titanium and aluminum atoms (Al_4_Ti_2_ and Al_2_Ti_4_), as shown in Figure 5c. Since the covalence of Ti-Al bond makes electrons aggregate between Ti and Al atoms, the Ti-O bond strength in the Al_4_Ti_2_ and Al_2_Ti_4_ octahedrons is higher than that in the Ti_6_ octahedron. Hence, the oxygen diffusion ability in the lattice of the γ phase is much weaker than that in the lattice of the α_2_ phase, leading to the higher oxidation activation energy of the Ti-46Al-2Cr-5Nb alloy than that of the Ti-24Al-15Nb-1.5Mo alloy. The above analysis manifests that the oxidation mechanism of the Ti-46Al-2Cr-5Nb alloy at Stage II is the same with the Ti-24Al-15Nb-1.5Mo alloy, namely, oxygen dissolution in the alloy. Besides, since the oxygen solubility in the γ phase is much lower than that in the α_2_ phase, the temperature range corresponding to Stage II for the Ti-46Al-2Cr-5Nb alloy (870–980 °C) is narrower than that for the Ti-24Al-15Nb-1.5Mo alloy (800–1020 °C).

#### 4.3.3. Accelerated Oxidation Stage (Stage III)

When the temperature was raised to Stage III (980–1280 °C for the Ti-46Al-2Cr-5Nb alloy and 1020–1400 °C for the Ti-24Al-15Nb-1.5Mo alloy), the two alloys exhibited accelerated oxidation behaviors and the oxidation activation energies are respectively 249.8 and 244.8 kJ/mol, which is close to the diffusion activation energies of O^2-^ and Ti^4+^ in TiO_2_ (234 kJ/mol [37] and 257 kJ/mol [38], respectively). It indicates that the oxidation mechanisms of the two alloys at this stage are mainly the growth of oxide scales dominated by TiO_2_. Since the diffusion rates of O^2−^ and Ti^4+^ in TiO_2_ are much lower than that of oxygen atom in the alloy, the rate-determining step at this stage is the diffusion of O^2−^ and Ti^4+^ in the oxide scale so that the oxidation activation energy is independent with the matrix phases of the alloys.

**Figure 6:**
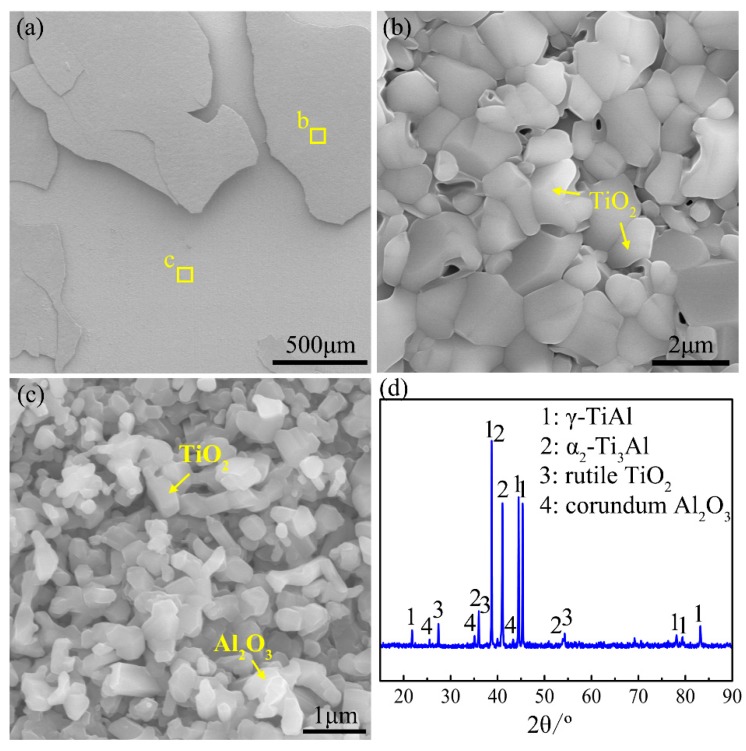
(**a**–**c**) Surface morphologies and (**d**) X-ray diffraction (XRD) pattern of the oxide formed during heating the Ti-46Al-2Cr-5Nb alloy to the end of Stage III.

Figure 6 demonstrates the surface morphologies and XRD pattern of the oxide scale formed during heating the Ti-46Al-2Cr-5Nb alloy to the end of Stage III. As shown in the XRD pattern (Figure 6d), there are high contents of γ and α_2_ phases besides rutile TiO_2_ and corundum α-Al_2_O_3_. It manifests that the oxide is composed of rutile TiO_2_ and corundum α-Al_2_O_3_ (hereafter referred to as TiO_2_ and Al_2_O_3_), and the thickness of the oxide scale is less than the detection depth of X-ray (~20 μm). The oxide scale is a multiple-layer structure (Figure 6a), the outer layer of which consists of coarse TiO_2_ crystals (Figure 6b) and is prone to peel off, exposing the inner layer of fine TiO_2_ and Al_2_O_3_ crystals (Figure 6c).

**Figure 7:**
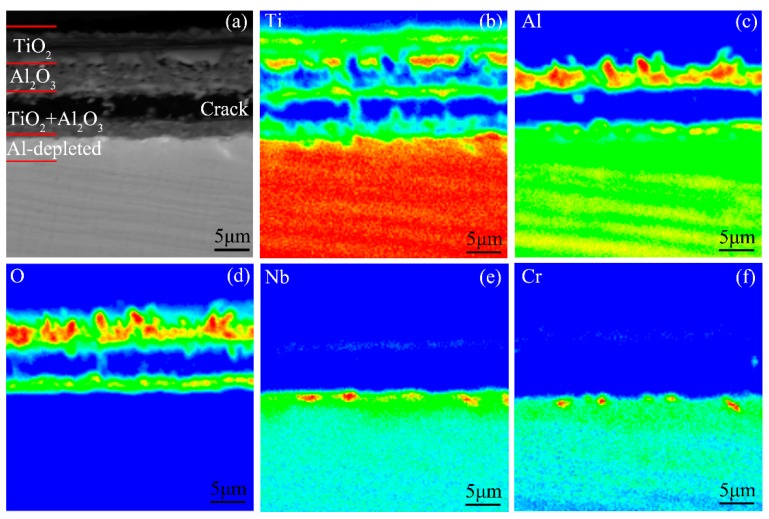
(**a**) Cross-sectional morphology and (**b**–**f**) the corresponding elemental distribution maps of the oxide scale formed during heating the Ti-46Al-2Cr-5Nb alloy to the end of Stage III.

Figure 7 presents the cross-sectional morphology and the corresponding elemental distribution maps of the oxide scale on the Ti-46Al-2Cr-5Nb alloy. The oxide scale is identified to be in the order of the TiO_2_ layer/Al_2_O_3_-rich layer/TiO_2_ + Al_2_O_3_ mixed layer from the outside to the inside. The total thickness of the oxide scale (~13 μm) is indeed less than the detection depth of X-ray, which is consistent with the result of the XRD pattern. An Al-depleted layer that deemed to be α_2_ phase [16] is generated in the subsurface of the alloy. Besides, there is a transverse crack between the intermediate and inner layers (Figure 7a). However, the crack is considered to be produced during preparation of the metallographic specimen instead of during oxidation and during cooling, since no such crack is found in the oxide scale formed during heating the alloy to the end of Stage IV and the alloy exhibited much severer oxidation behavior at Stage IV than at Stage III. Thus, the crack is not taken into account when discussing the formation process of the oxide scale at Stage III.

The oxide scale formed during heating the Ti-46Al-2Cr-5Nb alloy from room temperature to the end of Stage III (Figure 6 and Figure 7) is considered to be mainly generated at Stage III since the Ti-46Al-2Cr-5Nb alloy exhibits nearly non-oxidation behavior at Stage I and the oxidation mechanism at Stage II is oxygen dissolution in the alloy. The three-layer oxide scale structure formed at Stage III (980–1280 °C) is similar to the common oxide scale structure formed during isothermal oxidation at 800–1000 °C [14]. Hence, the growth mechanism of the oxide scale at Stage III could be deduced from the isothermal oxidation mechanism at 800–1000 °C. Firstly, an Al_2_O_3_ film is rapidly generated on the oxygen-saturated Ti-46Al-2Cr-5Nb alloy [39]. Since the Al_2_O_3_ film is grown by the inward diffusion of O^2−^ along the Al_2_O_3_ grain boundaries and the outward diffusion of Al^3+^ along the Al_2_O_3_ lattice, the Al_2_O_3_ grain boundaries suffer compressive stress, resulting in curling deformation or even rupture of the Al_2_O_3_ film [40]. Thus, Ti^4+^ and O^2-^ would diffuse in opposite directions along the cracks of the Al_2_O_3_ film [40]. As a result, an outer TiO_2_ layer and an inner TiO_2_ + Al_2_O_3_ mixed layer are respectively formed on the outside and the inside of the ruptured Al_2_O_3_ film, as shown in Figure 7. The growth process of the oxide scale at Stage III demonstrates that the growth rate of the oxide scale is mainly controlled by the diffusion of Ti^4+^ and O^2−^ in TiO_2_, which is consistent with the result of the oxidation activation energy of the alloy at Stage III. Therefore, it is further confirmed that the oxidation mechanism of the Ti-46Al-2Cr-5Nb alloy at Stage III is the growth of the oxide scale dominated by TiO_2_.

**Figure 8:**
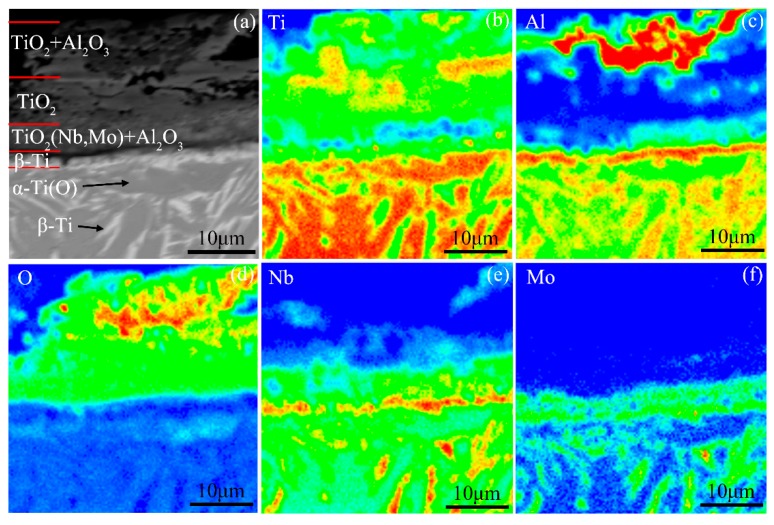
(**a**) Cross-sectional morphology and (**b**–**f**) the corresponding elemental distribution maps of the oxide scale formed during heating the Ti-24Al-15Nb-1.5Mo alloy to the end of Stage III.

As for the Ti-24Al-15Nb-1.5Mo alloy, the oxide scale formed during heating the alloy to the end of Stage III has a three-layer structure, which is in the order of the TiO_2_ + Al_2_O_3_ mixed layer/TiO_2_-rich layer/TiO_2_(Nb, Mo) + Al_2_O_3_ mixed layer from the outside to the inside, as shown in Figure 8. Consistent with the oxidation activation energy, the structure of the oxide scale also indicates that the oxidation mechanism of the Ti-24Al-15Nb-1.5Mo alloy is mainly the growth of the oxide scale dominated by TiO_2_. Besides, a thin β-Ti layer enriched in Al, Nb and Mo elements was formed in the subsurface of the alloy. The microstructure between the β-Ti layer and the matrix is composed of α-Ti(O) grains and a small amount of β-Ti phase enriched in Al, Nb, and Mo in the grain boundaries. As mentioned in Section 4.3, the matrix is dominated by β phase at the end of Stage III. Thus, it is referred that the microstructure between the subsurface of the alloy and the matrix is stabilized to α phase by the inward-diffusing oxygen, while the microstructure of the subsurface still maintains β phase due to the enrichment of Nb and Mo.

#### 4.3.4. Severe Oxidation Stage (Stage IV)

The two alloys exhibited severe oxidation behaviors when the temperature was raised to Stage IV, while the corresponding temperature range of the Ti-46Al-2Cr-5Nb alloy (1400–1450 °C) is much higher than that of the Ti-24Al-15Nb-1.5Mo alloy (1280–1350 °C). The oxidation activation energies of the Ti-46Al-2Cr-5Nb and Ti-24Al-15Nb-1.5Mo alloys are respectively 985.0 and 608.6 kJ/mol, which is significantly higher than those at Stage III (249.8 and 244.8 kJ/mol). Thus, the oxidation mechanisms at Stage IV should be somewhat different from that at Stage III.

**Figure 9:**
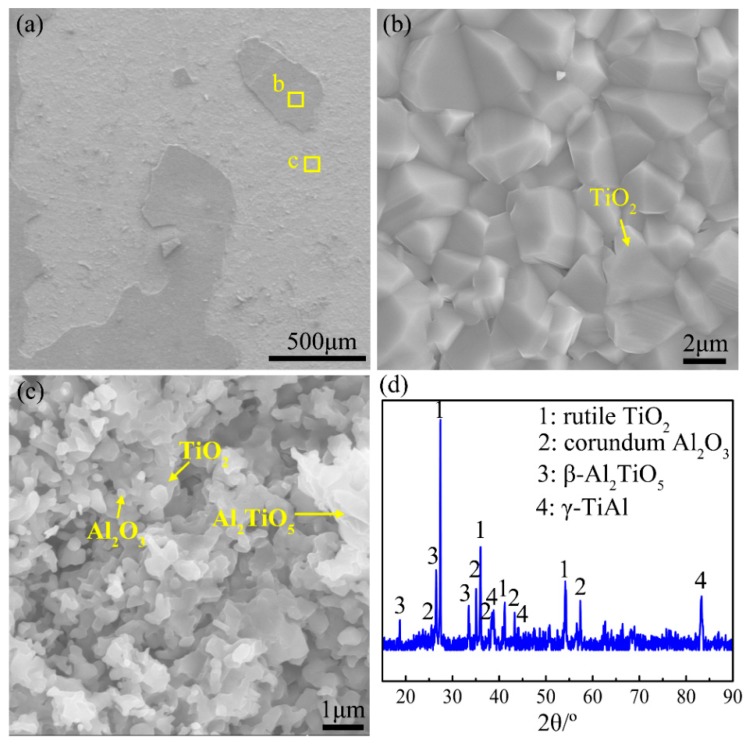
(**a**–**c**) Surface morphologies and (**d**) XRD pattern of the oxide scale formed during heating the Ti-46Al-2Cr-5Nb alloy to the end of Stage IV.

Figure 9 shows the surface morphologies and XRD pattern of the oxide scale formed during heating the Ti-46Al-2Cr-5Nb alloy to the end of Stage IV. As shown in the XRD pattern (Figure 9d), the oxide scale is mainly composed of a large amount of TiO_2_ as well as a small amount of Al_2_O_3_ and β-Al_2_TiO_5_. Besides, the content of the γ phase is much lower than that detected after heating to the end of Stage III (Figure 6d). It manifests that the thickness of the oxide scale increased significantly at Stage IV compared with that at Stage III, which is consistent with the drastic increase of the oxidation rate at Stage IV (Figure 3). As shown in Figure 9a,b, the outer layer of the oxide scale is still a spalling-prone TiO_2_ layer, but it is relatively thin and dense compared with that formed at Stage III (Figure 6a,b). Below the outer layer, a few irregular sintering structures of β-Al_2_TiO_5_ coexist with relatively regular crystals of TiO_2_ and Al_2_O_3_, as shown in Figure 9c. It can be seen from the TiO_2_-Al_2_O_3_ phase diagram [41] that the reaction of TiO_2_ + Al_2_O_3_ → β-Al_2_TiO_5_ occurs when the temperature is higher than 1200 °C. Therefore, the irregular sintering structures of β-Al_2_TiO_5_ was produced by the reaction between TiO_2_ and Al_2_O_3_ in the oxide scale at Stage IV (1280–1350 °C).

**Figure 10:**
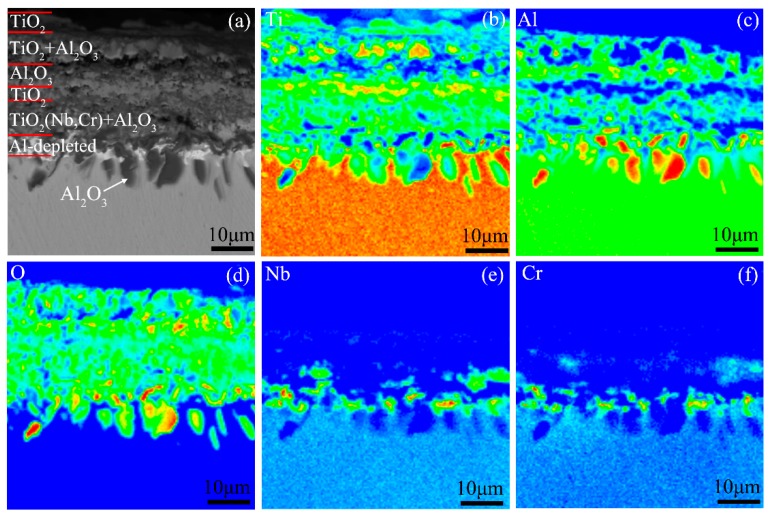
(**a**) Cross-sectional morphology and (**b***–***f**) the corresponding elemental distribution maps of the oxide scale formed during heating the Ti-46Al-2Cr-5Nb alloy to the end of Stage IV.

Figure 10 presents the cross-sectional morphology and the corresponding elemental distribution maps of the oxide scale. The oxide scale is identified to be in the order of the TiO_2_ layer/TiO_2_ + Al_2_O_3_ mixed layer/Al_2_O_3_-rich layer/TiO_2_-rich layer/TiO_2_(Nb, Cr) + Al_2_O_3_ mixed layer from the outside to the inside. In addition, an Al-depleted layer enriched in Nb and Cr elements is generated in the subsurface of the alloy. Moreover, Al_2_O_3_ oxides are dispersed as islands in the subsurface, indicating the occurrence of internal oxidation at Stage IV. Since internal oxidation deteriorates the oxidation resistance of TiAl-based alloys [14], the severe oxidation behavior of the Ti-46Al-2Cr-5Nb alloy at this stage resulted from the internal oxidation of Al, which is probably the cause for the much higher oxidation energy of the Ti-46Al-2Cr-5Nb alloy at Stage IV than that at Stage III.

**Figure 11:**
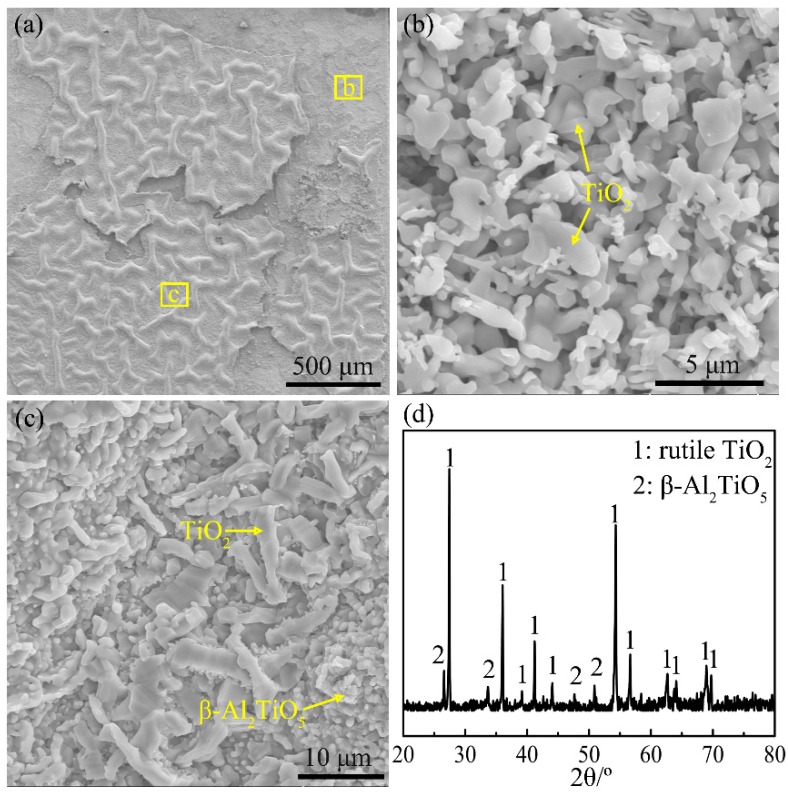
(**a**–**c**) Surface morphologies and (**d**) XRD pattern of the oxide formed during continuously heating the Ti-24Al-15Nb-1.5Mo alloy to the end of Stage IV.

As for the Ti-24Al-15Nb-1.5Mo alloy, the surface morphologies and XRD pattern of the oxide scale formed during heating the alloy to the end of Stage IV are shown in Figure 11. The XRD result shows that the oxide scale is mainly composed of TiO_2_ accompanied with a small amount of β-Al_2_TiO_5_ (Figure 11d). The outer layer of the oxide scale is crimping (Figure 11a), consisting of coarse rod-like TiO_2_ particles and ridged structures composed of fine β-Al_2_TiO_5_ particles (Figure 11c). The outer layer is prone to exfoliation (Figure 11a), revealing the layer of fine TiO_2_ particles (Figure 11b).

**Figure 12:**
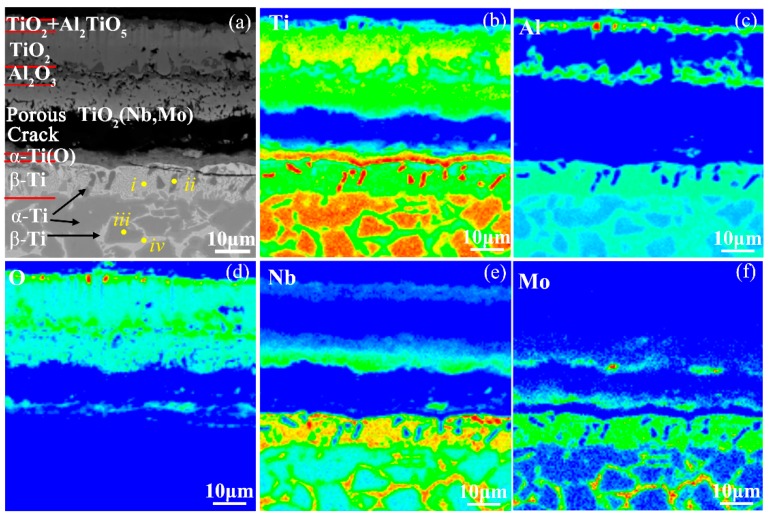
(**a**) Cross-sectional morphology and (**b**–**f**) the corresponding elemental distribution maps of the oxide scale formed during heating the Ti-24Al-15Nb-1.5Mo alloy to the end of Stage IV.

Figure 12 presents the cross-sectional morphology and the corresponding elemental distribution maps of the oxide scale on the Ti-24Al-15Nb-1.5Mo alloy. The structure of the oxide scale is in the order of the TiO_2_ + Al_2_TiO_5_ layer/TiO_2_ layer/coarse Al_2_O_3_ layer/porous TiO_2_(Nb, Mo) layer from the outside to the inside. The porous TiO_2_(Nb, Mo) inner layer and the coarse Al_2_O_3_ immediate layer indicate that the Al_2_O_3_ particles formed in the inner layer dissolved in the surrounding TiO_2_(Nb, Mo), then migrated outward and re-precipitated in the immediate layer at Stage IV, the reason for which is that the decreasing oxygen pressure around the inner layer with the thickening of the oxide scale increases the solubility of Al_2_O_3_ in TiO_2_ and reduces the stability of Al_2_O_3_ [17]. It is reported that the dissolution, migration and re-precipitation of Al_2_O_3_ destroys the oxygen-blocking ability of the original Al_2_O_3_ barrier layer, leading to breakaway oxidation [17,42]. However, there is a transverse crack below the oxide scale and whether the crack contributed to the severe oxidation should be discussed. According to the crimping structure and the locally spalling morphology of the oxide scale (Figure 11a), the crack is considered to be induced during cooling after oxidation. The reason is that the oxide scale and the matrix were respectively subjected to compressive and tensile stresses during cooling since the thermal expansion coefficient of the oxide scale is generally smaller than that of the metal, and curling deformation was prone to occur in the oxide scale for stress release. Thus, the severe oxidation behavior of the Ti-24Al-15Nb-1.5Mo alloy at Stage IV is independent of the formation of the transverse crack. Consequently, the severe oxidation at this stage is due to the dissolution, migration and re-precipitation of Al_2_O_3_, which might lead to the higher oxidation energy at Stage IV than that at Stage III.

In addition, a thin α-Ti(O) layer was formed at the interface between the oxide scale and the alloy, beneath which is a 10-μm-thick layer composed of β-Ti rich in Nb, Mo, Al elements (referred to as *i* in Figure 12a) and several fine α-Ti grains with an orientation nearly perpendicular to the matrix surface (referred to as *ii* in Figure 12a). The microstructure between the β-Ti layer and the matrix is still composed of α-Ti(O) grains (referred to as *iii* in Figure 12a) and a small amount of β-Ti phase in the grain boundaries (referred to as *iv* in Figure 12a). The chemical compositions of these structures are shown in Table 2. The microstructures demonstrate that more oxygen diffused into the subsurface of the alloy at Stage IV in comparison with at Stage III, resulting in the transition from β-Ti to α-Ti(O) in the near subsurface layer.

#### 4.3.5. Decelerated Oxidation Stage (Stage V)

**Figure 13:**
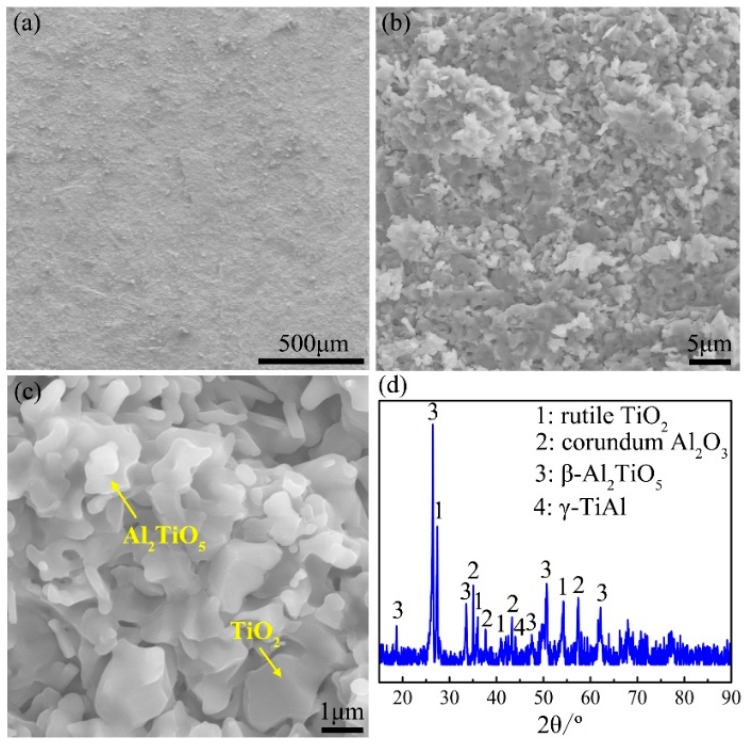
(**a**–**c**) Surface morphologies and (**d**) XRD pattern of the oxide scale formed during heating the Ti-46Al-2Cr-5Nb alloy to the end of Stage V.

When the temperature increased to Stage V (1350–1450 °C), the oxidation rate of the Ti-46Al-2Cr-5Nb alloy decreased remarkably. Figure 13 presents the surface morphologies and XRD pattern of the oxide scale formed during heating the Ti-46Al-2Cr-5Nb alloy to the end of Stage V. There is no obvious exfoliation morphology on the surface of oxide scale (Figure 13a). Further, the scale becomes denser (Figure 13b) and the crystals in the surface of the scale become larger (Figure 13c) compared with those formed at Stage IV. As shown in the XRD pattern (Figure 13d), the oxide scale consists of a large quantity of β-Al_2_TiO_5_ as well as some TiO_2_ and Al_2_O_3_, indicating that the sintering reaction between TiO_2_ and Al_2_O_3_ in the oxide scale aggravated at Stage V.

**Figure 14:**
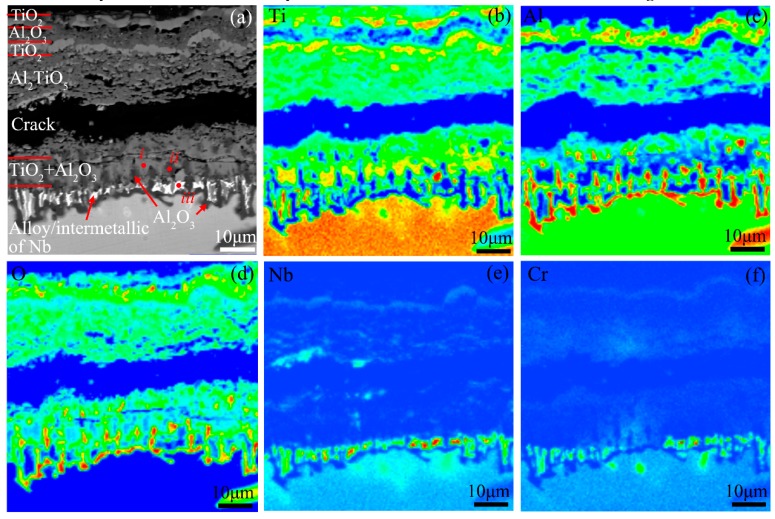
(**a**) Cross-sectional morphology and (**b**–**f**) the corresponding elemental distribution maps of the oxide scale formed during heating the Ti-46Al-2Cr-5Nb alloy to the end of Stage V.

Figure 14 shows the cross-sectional morphology and the corresponding elemental distribution maps of the oxide scale. The oxide scale from the outside to the inside is identified to be in the order of the TiO_2_ layer/Al_2_O_3_-rich layer/TiO_2_-rich layer/Al_2_TiO_5_ layer/mixed layer of TiO_2_ and fine Al_2_O_3_ flakes. It is interesting that the TiO_2_ (referred to as *i* in Figure 14a) and the fine Al_2_O_3_ flakes (referred to as *ii* in Figure 14a) distribute alternately in the inner layer, the compositions of which are presented in Table 3. White coarse particles (referred to as *iii* in Figure 14a) with an orientation perpendicular to the oxide/substrate interface were generated in the subsurface of the substrate. The detected composition as shown in Table 3 manifests that the white particles might be an alloy or an intermetallic of Nb. Moreover, there are a lot of fine Al_2_O_3_ flakes distributing around the white coarse particles. Besides, it should be mentioned that there is a transverse crack in the oxide scale, which is not found in the oxide scale formed during heating the alloy to the end of Stage IV. This crack is considered to be produced during cooling after oxidation or during the preparation of metallographic specimens instead of during oxidation, since the oxidation rate at Stage V was significantly reduced in comparison with at Stage IV.

Through comparing the cross-sectional structures of the oxide scales respectively formed by heating the Ti-46Al-2Cr-5Nb alloy to the end of Stage IV and Stage V (Figure 10 and Figure 14), the growth process of the oxide scale at Stage V can be inferred as follows: (1) oxygen from the atmosphere diffused into the alloy and the Al-depleted zones were preferentially oxidized nearby the Al_2_O_3_ flakes which were generated due to the internal oxidation at Stage IV, thus forming the inner oxide layer structure where TiO_2_ and Al_2_O_3_ flakes distributed alternately; (2) due to the limited solubility of the alloying elements (such as Nb, Cr and Al) in TiO_2_, the alloying elements diffused towards the underlying substrate during the growth of the oxide scale, resulting in the formation of massive white coarse particles of Nb alloy or intermetallic in the newly-formed subsurface of the substrate; (3) the continuous inward diffusion of oxygen further induced the occurrence of internal oxidation in the subsurface, leading to the formation of fine Al_2_O_3_ flakes around the white coarse particles and even the underlying substrate. However, at the same time of the oxide growth, the sintering reaction between TiO_2_ and Al_2_O_3_ in the oxide scale (mainly in the TiO_2_ + Al_2_O_3_ mixed layer) aggravated and an β-Al_2_TiO_5_-rich layer was formed in the oxide scale (Figure 14a). Since Al_2_TiO_5_ has higher oxygen resistance than TiO_2_ [5,43], the formation of the β-Al_2_TiO_5_-rich layer could effectively slow down the inward diffusion of oxygen and the oxidation rate decreased. Hence, the decelerated oxidation behavior of the Ti-46Al-2Cr-5Nb alloy at Stage V is due to the generation of an oxygen-barrier β-Al_2_TiO_5_-rich layer in the oxide scale by the reaction between TiO_2_ and Al_2_O_3_ in large scales.

### 4.4. Reasons for the Occurrence of Internal Oxidation in the Ti-46Al-2Cr-5Nb Alloy

In comparison with the Ti-24Al-15Nb-1.5Mo alloy, the Ti-46Al-2Cr-5Nb alloy suffered catastrophic oxidation at the temperature range of 1280–1350 °C due to the occurrence of internal oxidation. In order to improve the non-isothermal oxidation resistance of the Ti-46Al-2Cr-5Nb alloy, it is essential to shed light on the reasons for the occurrence of internal oxidation in the Ti-46Al-2Cr-5Nb alloy instead of in the Ti-24Al-15Nb-1.5Mo alloy. In accordance to the occurrence conditions of internal oxidation [44], it seems that the Ti-24Al-15Nb-1.5Mo alloy mainly dominated by α_2_ phase should be more prone to suffer internal oxidation than the Ti-46Al-2Cr-5Nb alloy mainly dominated by γ phase, since both the oxygen solubility and the oxygen diffusion rate in α_2_ phase are higher than those in the γ phase and the Al content in the α_2_ phase is lower than that in the γ phase. However, this is not the case. Therefore, the actual phases in the Ti-46Al-2Cr-5Nb and Ti-24Al-15Nb-1.5Mo alloys at the temperature range of 1280–1350 °C should be taken into account.

**Figure 15:**
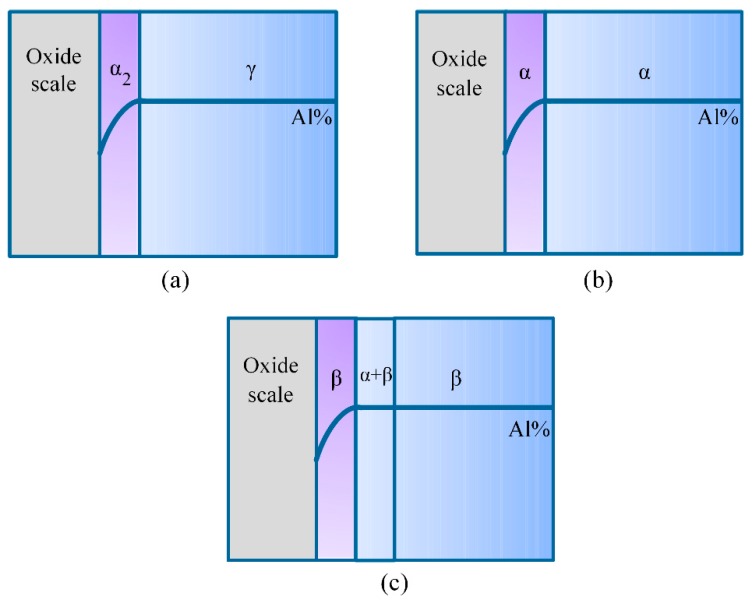
Schematic diagrams of the phase structures for (**a**) the Ti-46Al-2Cr-5Nb alloy when T < 1280 °C, (**b**) the Ti-46Al-2Cr-5Nb alloy when T > 1280 °C and (**c**) the Ti-24Al-15Nb-1.5Mo alloy at the temperature range of 1280–1350 °C.

The initial temperature for the occurrence of internal oxidation in the Ti-46Al-2Cr-5Nb alloy (1280 °C) is close to the temperature of γ + α → α phase transition (about 1226 °C, see Figure 3a). Hence, before the occurrence of internal oxidation (T < 1280 °C), the substrate of the Ti-46Al-2Cr-5Nb alloy was mainly dominated by γ phase and the subsurface of the alloy was dominated by an Al-depleted layer of α_2_ phase [16], as illustrated in Figure 15a. However, when the temperature rose above the initial temperature for the occurrence of internal oxidation (T > 1280 °C), the main phases both in the substrate and the subsurface Al-depleted layer of the Ti-46Al-2Cr-5Nb alloy transformed to the α phase, as illustrated in Figure 15b. As for the Ti-24Al-15Nb-1.5Mo alloy at the temperature range for the occurrence of internal oxidation in the Ti-46Al-2Cr-5Nb alloy (1280–1350 °C), the substrate was mainly dominated by β phase and the subsurface was also dominated by β phase due to the enrichment of Mo and Nb elements, even though the microstructure between the subsurface and the substrate was composed of α-Ti(O) grains and a small amount of β-Ti phase, as illustrated in Figure 15c. It is obviously seen from Figure 15 that the phases in the subsurface where internal oxidation occurs are different. Hence, in order to reveal the effect of the phases in the subsurface on the internal oxidation tendency, the four phases involving in the Ti-Al alloys (γ, α_2_, α and β) were studied.

The tendency of internal oxidation for the different phases in the Ti-46Al-2Cr-5Nb alloy at 1280 °C can be identified based on Wagner’s theory [44]. The critical criterion for the transition from internal oxidation to external oxidation is:

(2) $$N_{\mathrm{Al}}>N_{\mathrm{crit}}{}_{\left(\mathrm{Al}\right)}={\left(\frac{{\pi g}^{*}}{2}\frac{V_{m}}{V_{\mathrm{ox}}}\frac{D_{O}}{D_{\mathrm{Al}}}N_{O}\right)}^{0.5},$$

where *N*_crit(Al)_ is the critical Al content required for the transition from internal oxidation to external oxidation, g* is a constant factor in the case of an index of 0.3, *V*_m_ and *V*_ox_ are respectively the molar volumes of the Ti-46Al-2Cr-5Nb alloy and Al_2_O_3_ oxide (in cm^3^/mol, V_ox_ = 25.5 cm^3^/mol), *D*_O_ and *D*_Al_ are respectively the diffusion coefficients of O and Al atoms in the lattice of the alloy, *N*_O_ and *N*_Al_ are respectively the oxygen content and Al content in the subsurface of the alloy. The composition in the subsurface of the Ti-46Al-2Cr-5Nb alloy after heating to 1280 °C was detected to be Ti-29.8Al-3.6O-3.0Cr-5.4Nb (in at.%) by EPMA, thus the values for *N*_O_ and *N*_Al_ are respectively 0.036 and 0.298. The values for the other parameters involved in Equation (2) are given in Table 4. It should be noted that the specific value for the oxygen diffusion coefficient in the γ phase has not been reported, but it is readily inferred that the oxygen diffusion coefficient in the γ phase is lower than that in the α_2_ phase from their lattice structure differences, as mentioned in Section 4.3.2.

Table 4 presents the calculation results of the critical Al contents required for the transition from internal oxidation to external oxidation for the different phases in the subsurface of the Ti-46Al-2Cr-5Nb alloy at 1280 °C. The critical Al contents required for the α_2_ and γ phases are respectively 0.085 and less than 0.05, which is much lower than the actual Al content in the subsurface of the substrate (*N*_Al_ = 0.298). It indicates that if the subsurface of the Ti-46Al-2Cr-5Nb alloy is dominated by γ phase and/or α_2_ phase, an external oxide scale would form on the alloy and internal oxidation could not occur. However, the critical Al contents required for the α and β phases are respectively 2.08 and 1.62, which is impractical since the contents are more than 1. This situation is caused by the fact that Wagner’s theory of internal oxidation is based on a large number of idealized conditions that often could not be met in the actual system [44]. Nevertheless, Wagner’s critical criterion can still reflect the tendency of internal oxidation for the different phases in the subsurface of the Ti-46Al-2Cr-5Nb alloy. The higher the required critical Al content, the more easily internal oxidation will occur. Therefore, the tendency of internal oxidation for the phases is in the order of α > β > α_2_ > γ. Thus, it is easy to understand why internal oxidation occurred in the Ti-46Al-2Cr-5Nb alloy when the temperature exceeded 1280 °C, since the main phase in the subsurface of the Ti-46Al-2Cr-5Nb alloy changed from α_2_ phase to α phase when the temperature was higher than 1280 °C. In addition, the main phase in the substrate of the Ti-24Al-15Nb-1.5Mo alloy was β phase at the temperature range of 1280–1350 °C due to the enrichment of large amounts of β-stabilizing elements (Nb and Mo), so that no internal oxidation occurred in this alloy. Consequently, it is concluded that the formation of α phase in the subsurface is the basic reason for the occurrence of internal oxidation in the Ti-46Al-2Cr-5Nb alloy. The tendency of internal oxidation in the TiAl-based alloys could be reduced through avoiding the formation of α phase in the subsurface by optimizing alloy ingredients, such as increasing the Al content or adding β-stabilizing elements. However, it should be noted that the optimization of alloy ingredients is complicated and should be further investigated since not only the tendency of internal oxidation, but also other properties of the alloys such as the mechanical properties, should be taken into consideration in practical engineering.

## 5. Conclusions

1. The non-isothermal oxidation behaviors of the Ti-46Al-2Cr-5Nb and Ti-24Al-15Nb-1.5Mo alloys are similar when the temperature is below 1280 °C, while the Ti-46Al-2Cr-5Nb alloy exhibits poorer oxidation resistance than the Ti-24Al-15Nb-1.5Mo alloy when the temperature exceeds 1280 °C, even though the oxidation rate of the Ti-46Al-2Cr-5Nb alloy decreases significantly when the temperature is above 1350 °C.
2. There are five stages in the non-isothermal oxidation process of the Ti-46Al-2Cr-5Nb alloy, including nearly non-oxidation (<870 °C), slow oxidation (870–980 °C), accelerated oxidation (980–1280 °C), severe oxidation (1280–1350 °C) and decelerated oxidation (1350–1450 °C) stages. The corresponding oxidation mechanisms are as follows: oxygen-barrier effect of the thin titanium oxide film; oxygen dissolution in the alloy; growth of the oxide scale dominated by TiO_2_; internal oxidation of Al; formation of an oxygen-barrier Al_2_TiO_5_-rich layer by reaction between TiO_2_ and Al_2_O_3_ in the oxide scale.
3. There are four stages in the non-isothermal oxidation process of the Ti-24Al-15Nb-1.5Mo alloy, including nearly non-oxidation (<800 °C), slow oxidation (800–1020 °C), accelerated oxidation (1020–1400 °C) and severe oxidation (1400–1450 °C) stages. The oxidation mechanisms for the first three stages are the same with that of the Ti-46Al-2Cr-5Nb alloy, while the oxidation mechanism for the last stage is the dissolution, migration and re-precipitation of Al_2_O_3_ in the oxide.
4. The tendency of internal oxidation for the different phases in the subsurface of the Ti-46Al-2Cr-5Nb alloy is in the order of α > β > α_2_ > γ. The formation of α phase in the subsurface is the basic reason for the occurrence of internal oxidation in the Ti-46Al-2Cr-5Nb alloy. The tendency of internal oxidation in the TiAl-based alloys could be reduced through avoiding the formation of α phase by optimizing alloy ingredients such as increasing Al content or adding β-stabilizing elements.

## Figures and Tables

**Table 1:** Temperature ranges, oxidation activation energies and matrix phases corresponding to different stages of the non-isothermal oxidation for the Ti-46Al-2Cr-5Nb and Ti-24Al-15Nb-1.5Mo alloys.

Stage	Temperature Range (°C)		Activation Energy (kJ/mol)		Matrix Phases	
	TiAl ^1^	Ti_3_Al ^2^	TiAl ^1^	Ti_3_Al ^2^	TiAl ^1^	Ti_3_Al ^2^
I	<870	<800	-	-	γ + α_2_	α_2_ + O + B2
II	870–980	800–1020	217.8	143.2	γ + α_2_	α_2_ + O + B2 → α_2_ + B2
III	980–1280	1020–1400	249.8	244.8	γ + α_2_ → γ + α→α	α_2_ + B2 → B2 → β
IV	1280–1350	1400–1450	985.0	608.6	α	β
V	1350–1450	-	-	-	α	-

^1^ TiAl here refers to the Ti-46Al-2Cr-5Nb alloy. ^2^ Ti_3_Al here refers to the Ti-24Al-15Nb-1.5Mo alloy.

**Table 2:** Chemical compositions of the microstructures in the Ti-24Al-15Nb-1.5Mo alloy examined by an electron probe microanalyzer (EPMA) after heating the alloy to the end of Stage IV.

Microstructures in Figure 12a	Compositions (at.%)			
	Ti	Al	Nb	Mo
*i*	42.99	20.15	34.66	2.2
*ii*	87.35	4.95	7.7	-
*iii*	75.62	12.77	11.23	0.37
*iv*	42.67	19.57	34.77	2.99

**Table 3:** Chemical compositions of the microstructures in the inner layer of the oxide and in the subsurface of the Ti-46Al-2Cr-5Nb alloy examined by EPMA after heating the alloy to the end of Stage IV.

Microstructures in Figure 14a	Compositions (at.%)				
	Ti	Al	Nb	Cr	O
*i*	44.61	3.96	0.24	-	51.19
*ii*	5.66	26.09	-	-	68.25
*iii*	29.25	14.25	39.59	16.91	-

**Table 4:** Critical Al contents required for the transition from internal oxidation to external oxidation for the different phases in the subsurface of the Ti-46Al-2Cr-5Nb alloy at 1280 °C.

Phases	Parameters				Results
	*V*_m_/(cm^3^/mol)	*D*_O_/(m^2^/s)	*D*_Al_ [45]/(m^2^/s)	*D*_O_/*D*_Al_	*N*_crit(Al)_
γ	19.7	<3.66 × 10^−15^	1.97 × 10^−14^	<0.186	<0.05
α_2_	40.7	3.66 × 10^−15^ [32]	1.37 × 10^−14^	0.267	0.085
α	10.6	3.54 × 10^−11^ [46]	5.80 × 10^−14^	610.3	2.08
β	11.0	3.75 × 10^−10^ [46]	1.04 × 10^−12^	360.6	1.62
